# Supplementary material for: Deletion of wfs1 Impairs Oligodendrocyte Precursor Cells Dorsal Distribution and Myelination Through the wfs1-hmgcs1 Axis in Zebrafish
Source: Biology (Basel). 2026 Aug 16;15(16):1402. doi: 10.3390/biology15161402 (PMC13510151; doi:10.3390/biology15161402)
Supplement: Supplementary file 1 [file biology-15-01402-s001.zip › File S1-Transcriptome sequencing data and differentially expressed genes (DEGs) in wfs1-- zebrafish at 5 dpf/2.KEGG/wfs1KOvsWT/ALL/src/dre04120.html]

dre04120


- dre:447935

- Down regulated genes

447935\_1(-1.51046996397832)

- dre:192341

- Up regulated genes

192341(1.80505057402621)

- dre:563708

- Down regulated genes

563708(-3.59233336704936)

- dre:323951

- Up regulated genes

323951\_2(1.8936939174037)

- Down regulated genes

323951(-1.8986634324385)
- dre:100151173

- Up regulated genes

100151173\_1(3.65905713487684)

- dre:559981

- Up regulated genes

559981(4.29260909627283)

- dre:692325

- Up regulated genes

692325(2.8401869052706)

- Down regulated genes

692325\_1(-2.68426544876755)

- dre:393223

- Down regulated genes

393223\_1(-2.70117970213935)

- dre:100136859

- Down regulated genes

100136859(-1.45934229812074)

- dre:192341

- Up regulated genes

192341(1.80505057402621)

- dre:447935

- Down regulated genes

447935\_1(-1.51046996397832)

- dre:323883

- Down regulated genes

323883(-4.8491500403135)

- dre:327215

- Down regulated genes

327215\_2(-4.79538777432529)

- dre:564149

- Up regulated genes

564149\_1(2.17670667568913)

- Down regulated genes

564149(-3.79984166474366)

- dre:373107

- Down regulated genes

373107\_1(-7.07514976036473)

- dre:556277

- Up regulated genes

556277(2.56614816386233)

- dre:393445

- Up regulated genes

393445\_1(1.1201391392804)

- dre:30100

- Down regulated genes

30100(-1.08477916155828)

- dre:100001302

- Down regulated genes

100001302(-1.47821568115234)

- dre:564866

- Up regulated genes

564866\_1(1.06162213658661)

- dre:559439

- Up regulated genes

559439\_1(1.8409908106629)

- dre:550134

- Up regulated genes

550134\_1(1.8446004641876)

- Down regulated genes

550134(-4.18593720111435)
- dre:777766

- Up regulated genes

novel.905(1.76535773054974)

Close
